# Supplementary material for: Longitudinal Patterns of Digital Parenting Restrictions and Adolescent Screen Use: Insights from the Adolescent Brain Cognitive Development (ABCD) Study
Source: medRxiv. 2025 Nov 25:2025.11.23.25340847. Preprint. [Version 1] doi: 10.1101/2025.11.23.25340847 (PMC12676394; doi:10.1101/2025.11.23.25340847)
Supplement: 1 [file NIHPP2025.11.23.25340847V1-supplement-1.pdf]

medRxiv preprint doi: https://doi.org/10.1101/2025.11.23.25340847; this version posted November 25, 2025. The copyright holder for this preprint (which was not certified by peer review) is the author/funder, who has granted medRxiv a license to display the preprint in perpetuity.

It is made available under a CC-BY 4.0 International license .

Table S1. Reliability Estimates (McDonald's Omega) for PSQ Items Across Years 3, 4, and 5

medRxiv preprint doi: https://doi.org/10.1101/2025.11.23.25340847; this version posted November 25, 2025. The copyright holder for this preprint (which was not certified by peer review) is the author/funder, who has granted medRxiv a license to display the preprint in perpetuity.

It is made available under a CC-BY 4.0 International license .

	McDonald's Omega (ω)		
	β	SE	p-value
**Visit**			
Year-3	0.918	0.001	<0.001
Year-4	0.929	0.001	<0.001
Year-5	0.933	0.001	<0.001

Table S2. Final Class Counts and Proportions for Latent Class Trajectories Across Years 3, 4, and 5

Year 3	Year 4	Year 5	Count	Proportion (%)

medRxiv preprint doi: https://doi.org/10.1101/2025.11.23.25340847; this version posted November 25, 2025. The copyright holder for this preprint (which was not certified by peer review) is the author/funder, who has granted medRxiv a license to display the preprint in perpetuity.

It is made available under a CC-BY 4.0 International license .

**High**	**High**	**High**	**1,739.0**	**21.5%**
High	High	Moderate	633.0	7.8%
High	High	Low	230.0	2.8%
High	Moderate	High	55.0	0.7%
High	Moderate	Moderate	556.0	6.9%
High	Moderate	Low	250.0	3.1%
High	Low	High	30.0	0.4%
High	Low	Moderate	67.0	0.8%
High	Low	Low	236.0	2.9%
Moderate	High	High	89.0	1.1%
Moderate	High	Moderate	75.0	0.9%
Moderate	High	Low	14.0	0.2%
Moderate	Moderate	High	56.0	0.7%
**Moderate**	**Moderate**	**Moderate**	**1,443.0**	**17.8%**
Moderate	Moderate	Low	693.0	8.6%
Moderate	Low	High	22.0	0.3%
Moderate	Low	Moderate	172.0	2.1%
Moderate	Low	Low	565.0	7.0%
Low	High	High	38.0	0.5%
Low	High	Moderate	11.0	0.1%
Low	High	Low	20.0	0.2%
Low	Moderate	High	3.0	0.0%
Low	Moderate	Moderate	80.0	1.0%
Low	Moderate	Low	106.0	1.3%
Low	Low	High	12.0	0.1%
Low	Low	Moderate	89.0	1.1%
**Low**	**Low**	**Low**	**805.0**	**10.0%**

Note. High, Moderate, and Low refer to restriction levels.

Table S3. Latent Transition Analysis Model Fit Indices for Year 3 to Year 5 (n=8096)

medRxiv preprint doi: https://doi.org/10.1101/2025.11.23.25340847; this version posted November 25, 2025. The copyright holder for this preprint (which was not certified by peer review) is the author/funder, who has granted medRxiv a license to display the preprint in perpetuity.

It is made available under a CC-BY 4.0 International license .

Number of Classes	LL	BIC	SABIC	CAIC	Entropy	Smallest off-diagonal ALCPP	Average latent class posterior probabilities
2	-368100	737002	736719	736379	0.868	0.936	0.972, 0.936
3	-352149	705557	705112	704578	0.876	0.919	0.949, 0.919, 0.933
4	-343633	689030	688407	687658	0.886	0.923	0.923, 0.933, 0.944, 0.923
5	-339385	681064	680254	679280	0.875	0.881	0.881. 0.889, 0.937, 0.91, 0.993
6	-335460	673782	672772	671557	0.871	0.871	0.871, 0.894, 0.933, 0.904, 0.888, 0.900

Note. LL = Log-Likelihood, CAIC = Consistent Akaike Information Criterion, BIC = Bayesian Information Criterion, SABIC = Sample-Size Adjusted Bayesian Information Criterion, ALCPP = Average Latent Class Posterior Probability

Table S4. Fit Statistics and Class Enumeration for Latent Class Analysis by Time Point

Model	# of classes	LL	BIC	SABI C	CAIC	Entro py

medRxiv preprint doi: https://doi.org/10.1101/2025.11.23.25340847; this version posted November 25, 2025. The copyright holder for this preprint (which was not certified by peer review) is the author/funder, who has granted medRxiv a license to display the preprint in perpetuity.

It is made available under a CC-BY 4.0 International license .

Year-3 visit (T1) (n=7954)	1	-128006	256390	256257	256097	NA
	2	-120389	241543	241273	240949	0.816
	3	-116047	233245	232838	232351	**0.882**
	4	-113355	228247	227703	227053	0.881
	5	-112144	226211	225531	224717	0.864
	6	-111228	224766	223949	222971	0.866
Year-4 visit (T2) (n=7920)	1	-131956	264289	264156	263996	NA
	2	-123426	247615	247345	247022	0.856
	3	-118242	237633	237226	236740	0.871
	4	-115641	232817	232274	231624	**0.889**
	5	-114447	230816	230136	229323	0.873
	6	-113348	229004	228188	227211	0.871
Year-5 visit (T3) (n=7891)	1	-134229	268836	268703	268543	NA
	2	-124599	249962	249691	249369	0.924
	3	-120104	241357	240951	240465	**0.873**
	4	-117998	237531	236988	236339	0.886
	5	-116630	235181	234501	233689	0.879
	6	-115501	233309	232492	231516	0.868

Note. LL = Log-Likelihood, CAIC = Consistent Akaike Information Criteria, BIC = Bayesian Information Criterion, SABIC = Sample-Size Adjusted Bayesian Information Criterion

medRxiv preprint doi: https://doi.org/10.1101/2025.11.23.25340847; this version posted November 25, 2025. The copyright holder for this preprint (which was not certified by peer review) is the author/funder, who has granted medRxiv a license to display the preprint in perpetuity.

It is made available under a CC-BY 4.0 International license .

Table S5. Fit Indices for Latent Transition Analysis Models With and Without Parameter Restrictions

	# of parameters	LL	CAIC	BIC	SS-ABIC	Entropy	Diagonal Average Latent Class Probabilities
Model with equality constraints	140	-361784	723848	724832	724388	0.873	0.917, 0.949, 0.933
Model without equality constraints	3416	-326152	659136	683042	672186	0.93	0.938, 0.929, 0.944

Note. The model without equality constraints failed to converge due to numerical instability, leading to potential local maxima and unreliable results.

LL = Log Likelihood, CAIC = Constant Akaike Information Criterion, BIC = Bayesian Information Criterion, SS-ABIC = Sample Size Adjusted Bayesian Information Criterion

medRxiv preprint doi: https://doi.org/10.1101/2025.11.23.25340847; this version posted November 25, 2025. The copyright holder for this preprint (which was not certified by peer review) is the author/funder, who has granted medRxiv a license to display the preprint in perpetuity.

It is made available under a CC-BY 4.0 International license .

Table S6. Frequency of Parent Responses to the ABCD Parent Screen Time Questionnaire (Years 3-5)

	**Strongly Disagree**	**Somewhat Disagree**	**Somewhat Agree**	**Strongly Agree**
1. When I am with my child, I use a screen-based device.	2376 (10%)	3557 (15%)	**11949 (50.5%)**	5793 (24.5%)
2. I try to limit how much I use a screen-based device when I am with my child	1507 (6.4%)	2090 (8.8%)	9929 (42%)	**10136 (42.8%)**
3. Our family often watches a screen during meals	**10689 (45.1%)**	4462 (18.8%)	5447 (23%)	3117 (13.1%)
4. Family members are allowed to use screen-based devices during meals	**12477 (52.6%)**	4690 (19.8%)	4568 (19.3%)	1977 (8.3%)
5. My child falls asleep while using a screen-based device	**13445 (56.7%)**	3829 (16.2%)	4527 (19.1%)	1899 (8.0%)
6. A screen-based device is usually playing in the room when my child falls asleep	**14351 (60.6%)**	3167 (13.4%)	3986 (16.8%)	2190 (9.2%)
7. My child has access to a mobile screen-based device in bed	**8596 (36.3%)**	2291 (9.7%)	5968 (25.2%)	6829 (28.8%)
8. I offer screen time to my child as a reward for good behavior	**13062 (46.1%)**	3681 (16.0%)	5013 (27.2%)	1841 (10.6%)
9. I take away screen time from my child as a punishment for bad behavior	4881 (20.7%)	2081 (8.8%)	7129 (30.3%)	**9448 (40.1%)**
10. I keep track of my child's screen time during the week	5226 (22.1%)	4693 (19.9%)	**8217 (34.8%)**	5479 (23.2%)
11. I keep track of my child's screen time during the weekend	6174 (26.1%)	5680 (24.0%)	**7453 (31.5%)**	4336 (18.3%)
12. I limit my child's screen time during the week.	4489 (19.0%)	3640 (15.4%)	8311 (35.1%)	**7215 (30.5%)**
13. I limit my child's screen time during the weekend.	6013 (25.4%)	6331 (26.8%)	**7235 (30.6%)**	4080 (17.2%)
14. I encourage my child to do activities other than screen time.	797 (3.4%)	387 (1.6%)	3997 (17.0%)	**18277 (77.9%)**

medRxiv preprint doi: https://doi.org/10.1101/2025.11.23.25340847; this version posted November 25, 2025. The copyright holder for this preprint (which was not certified by peer review) is the author/funder, who has granted medRxiv a license to display the preprint in perpetuity.

It is made available under a CC-BY 4.0 International license .

Note. Frequencies represent combined responses across years. Percentages represent proportion of response type by item.

Table S7. Conditional Probabilities of Class Membership at Year 5 Based on Year 3 and 4

Year 3 → Year 4	Probability of Low Restriction at Year 5	Probability of Moderate Restriction at Year 5	Probability of High Restriction at Year 5
Low → Low Restriction	**0.793**	0.174	0.032
Low → Moderate Restriction	0.323	**0.638**	0.039
Low → High Restriction	**0.101**	0.26	0.639
Moderate → Low Restriction	**0.793**	0.174	0.032
Moderate → Moderate Restriction	0.323	**0.638**	0.039
Moderate → High Restriction	0.101	0.26	**0.639**
High → Low Restriction	**0.793**	0.174	0.032
High → Moderate Restriction	0.323	**0.638**	0.039
High → High Restriction	0.101	0.26	**0.639**

Note: The table presents the conditional probabilities of class membership at Year 5 given transitions from Year 3 to Year 4. Cells with higher probabilities indicate the most likely class progression.

medRxiv preprint doi: https://doi.org/10.1101/2025.11.23.25340847; this version posted November 25, 2025. The copyright holder for this preprint (which was not certified by peer review) is the author/funder, who has granted medRxiv a license to display the preprint in perpetuity.

It is made available under a CC-BY 4.0 International license .

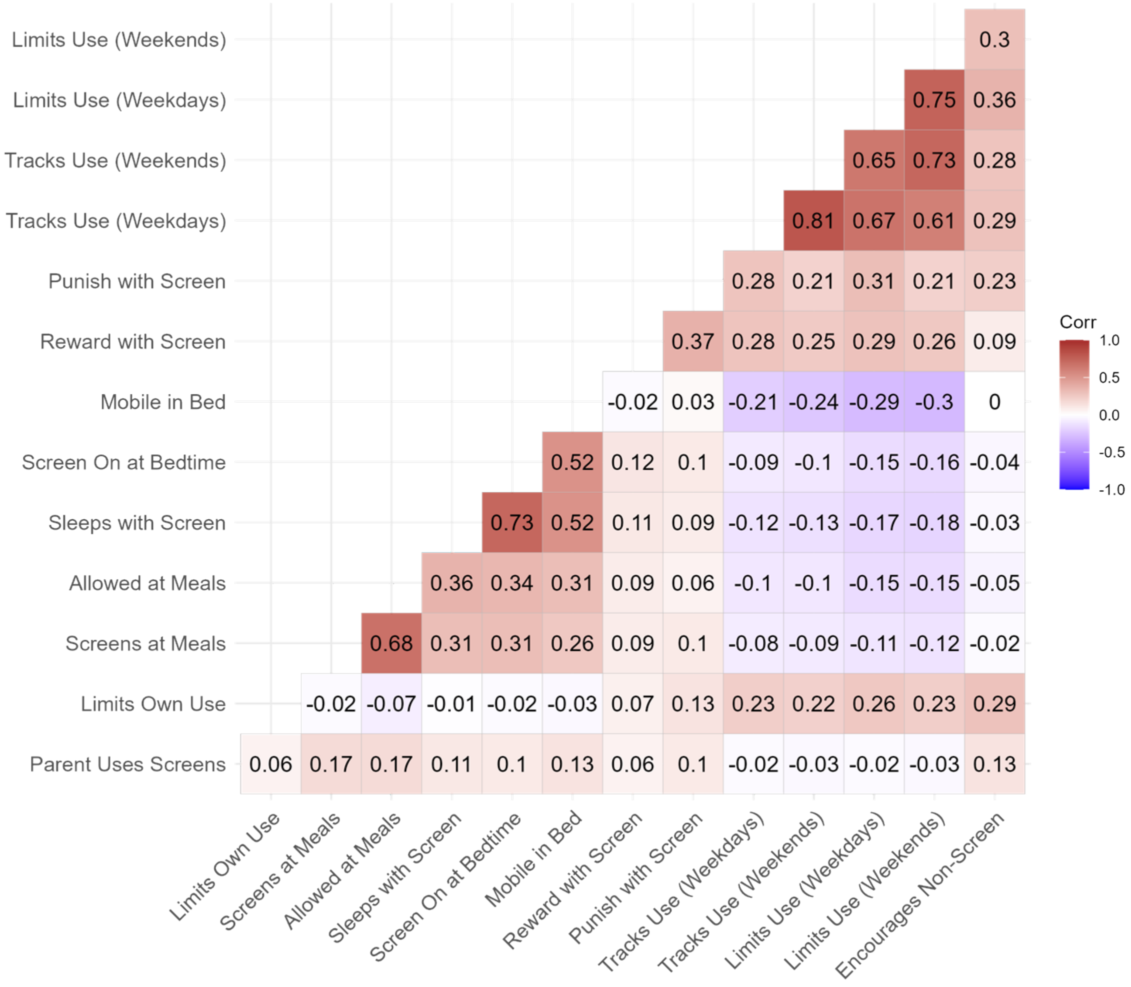

Figure S1. Correlation matrix of parental screen-related behaviors (PSQ items).

medRxiv preprint doi: https://doi.org/10.1101/2025.11.23.25340847; this version posted November 25, 2025. The copyright holder for this preprint (which was not certified by peer review) is the author/funder, who has granted medRxiv a license to display the preprint in perpetuity.

It is made available under a CC-BY 4.0 International license .

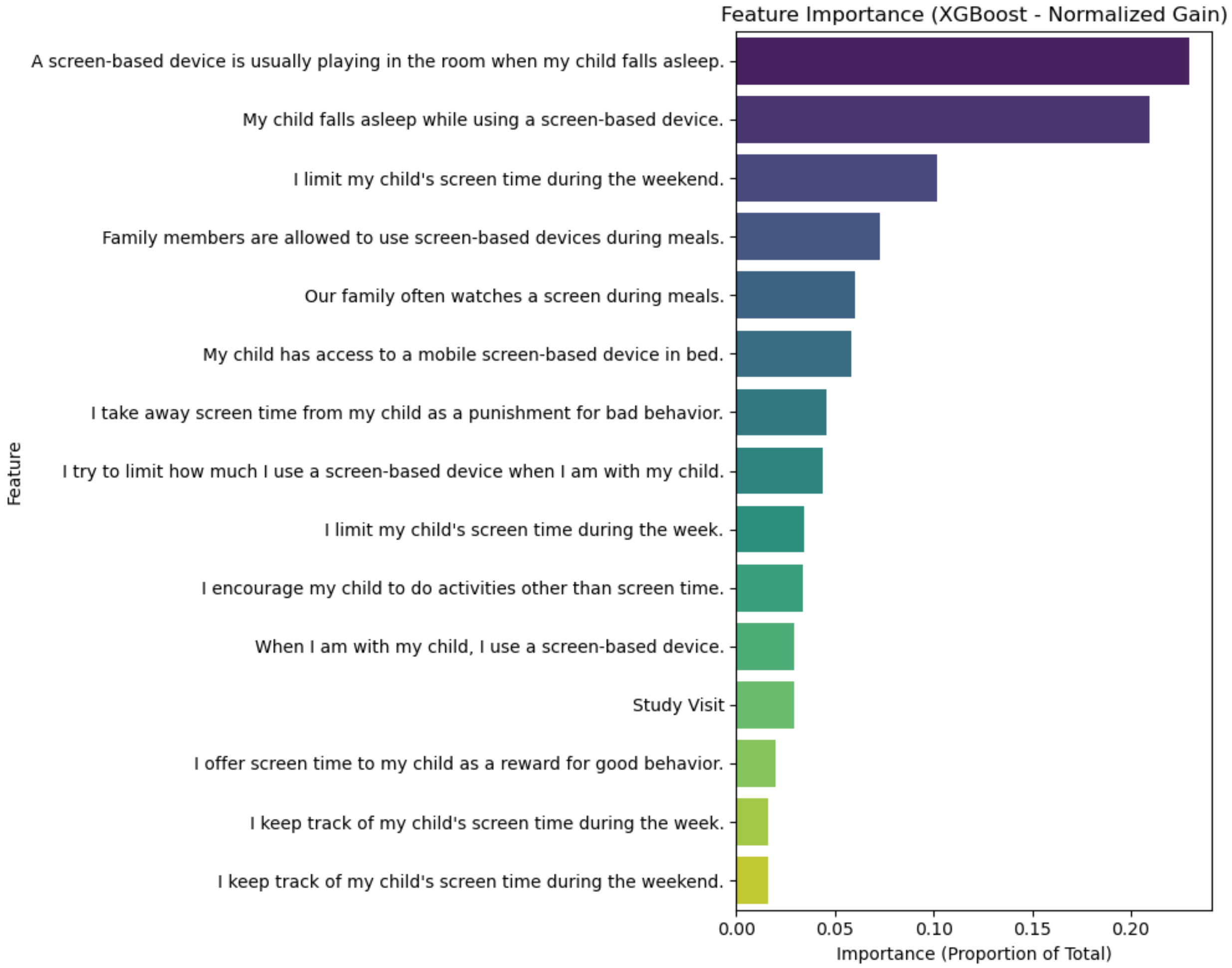

Figure S2. Feature Importance of Parenting Practices in Predicting Self-Reported Screen Time (XGBoost)
XGBoost-derived feature importance (Gain) for parenting practices predicting self-reported total screen time. Higher Gain values indicate greater contribution to model predictions.

medRxiv preprint doi: https://doi.org/10.1101/2025.11.23.25340847; this version posted November 25, 2025. The copyright holder for this preprint (which was not certified by peer review) is the author/funder, who has granted medRxiv a license to display the preprint in perpetuity.

It is made available under a CC-BY 4.0 International license .

Table S8. Model Performance Metrics for Training and Test Sets

**Metric**	**Training Set**	**Test Set**
R^2	0.146	0.102
Root Mean Squared Error	172.7	176.9
Mean Absolute Error	124.5	128.4

Note: R^2 = The proportion of variance explained by the model
